# A Robust Road Vanishing Point Detection Adapted to the Real-world Driving Scenes

**DOI:** 10.3390/s21062133

**Published:** 2021-03-18

**Authors:** Cuong Nguyen Khac, Yeongyu Choi, Ju H. Park, Ho-Youl Jung

**Affiliations:** 1 Department of Information and Communication Engineering, Yeungnam University, Gyeongsan 38544, Korea; cuongnguyenkhac73@ynu.ac.kr (C.N.K.); ygchoi11@ynu.ac.kr (Y.C.); 2 Department of Electrical Engineering, Yeungnam University, Gyeongsan 38544, Korea; jessie@ynu.ac.kr

**Keywords:** VP detection, optical flow, autonomous vehicles, ADAS, FOE, RANSAC

## Abstract

Vanishing point (VP) provides extremely useful information related to roads in driving scenes for advanced driver assistance systems (ADAS) and autonomous vehicles. Existing VP detection methods for driving scenes still have not achieved sufficiently high accuracy and robustness to apply for real-world driving scenes. This paper proposes a robust motion-based road VP detection method to compensate for the deficiencies. For such purposes, three main processing steps often used in the existing road VP detection methods are carefully examined. Based on the analysis, stable motion detection, stationary point-based motion vector selection, and angle-based RANSAC (RANdom SAmple Consensus) voting are proposed. A ground-truth driving dataset including various objects and illuminations is used to verify the robustness and real-time capability of the proposed method. The experimental results show that the proposed method outperforms the existing motion-based and edge-based road VP detection methods for various illumination conditioned driving scenes.

## 1. Introduction

If a set of lines that are parallel in the 3D object space is projected onto a 2D image plane of a camera under perspective view, their images will form a set of lines that intersect at a point. The intersection point is called a vanishing point (VP) [1]. Generally, the number of VPs in an image depends on the number of sets of parallel lines in the 3D object space that are projected on the image. Among a lot of VPs presented in every frame of the driving videos, the most special VP is the intersection point of lanes or road boundaries. Hereafter, the VP is called R-VP (road VP) [2] in the rest of this paper.

R-VPs provide important information regarding driving scenes for ADAS (advanced driver assistance systems) and autonomous vehicles. For example, camera rotations [3], depth distance estimations, an instantaneous moving direction of a host vehicle, drivable areas of roads, etc., can be determined by using R-VPs.

Recently, deep learning (DL) has achieved a lot of impressive successes. Particularly, CNN (convolutional neural networks) has obtained great results in image classification, image recognition, etc. Some studies have applied DL to R-VP detection. CNN is used to train R-VP detectors in [4,5,6]. Regression ResNet-34 is used as an R-VP detector in [7]. A combination of CNN and heatmap regression is applied to R-VP detection, where features get from a modified version of HRNet [8]. These DL-based R-VP detectors detect R-VPs for limited driving situations. Moreover, the supervised learning method in general is the data-driven learning method that needs a huge size of training datasets with the exact labels in real-world driving scenes. As can be seen in [4,5,6,7,8], the number of images in the training datasets is limited. It is still difficult to realize a sophisticated DL-based R-VP detection that is suitable for driving scenes in practice.

Currently, geometrical feature-based R-VP detection methods for driving scenes are still being studied and utilized. Recently, the number of geometrical feature-based methods for R-VP detection increased significantly. These recent existing methods can be classified into four groups based on types of geometrical features used in the methods, such as line segment-based [9,10,11,12,13,14,15,16,17,18,19,20,21,22,23,24,25,26,27,28,29], edge-based [30,31,32,33], motion-based [34,35,36,37], and texture-based methods [38,39,40,41,42,43,44,45,46,47,48,49,50,51,52].

Most geometrical feature-based R-VP detection methods include three main processing steps: Step (1) Line segments are extracted from various features in driving images. Step (2) The line segments that tend to converge at R-VP locations are selected. Step (3) Intersections of the selected line segments are used to vote R-VPs through a voting process.

Optical flows of image points are generated by the movement of an observer that is a camera of a host vehicle in the R-VP detection problem. The convergence point of the optical flows of stationary image points is called the focus of expansion (FOE) of the host vehicle [53]. The great advantage of the FOE is that the FOE forms from the self-movements of the host vehicle. In other words, the FOE formation of a host vehicle does not depend on geometrical feature extractions from lanes, road boundaries, and other lines. Furthermore, if a host vehicle moves in parallel with road boundaries and the roads are almost flat, its FOE will coincide with R-VP [2]. That is why FOEs of host vehicles can be used to detect efficiently R-VPs for both structured roads and unstructured roads in the existing motion-based R-VP detection methods. This paper carefully examines the main processing steps of the existing motion-based R-VP detection methods. Some disadvantages in these processing steps are analyzed and considered to design our proposed method. In this paper, a new motion-based R-VP detection method is proposed to overcome the shortcomings of the existing motion-based methods.

The novel contributions of this paper are outlined below.

1. Stable motion vector detection is proposed to detect the stable vectors from several consecutive frames.
2. Stationary point-based motion vector selection is proposed to keep useful motion vectors and to reduce unhelpful ones for the R-VP detection.
3. Angle-based RANSAC voting is proposed to reduce efficiently the contributions of the outliers.
4. The proposed R-VP detection method that consists of stable motion detection, stationary point-based motion vector selection, and angle-based RANSAC (RANdom SAmple Consensus) voting achieves high accuracy and robustness for real-world driving scenes.

The next section discusses related research work. Section 3 explains the proposed method in detail. Section 4 shows experimental results. We conclude in Section 5.

## 2. Related Work

### 2.1. Main Processing Steps of The Existing R-VP Detection Methods

Most existing R-VP detection methods consist mainly of three steps. In Step 1, the line segment-based methods extract line segments from lane markers and road boundaries. The edge-based methods and texture-based methods extract pieces that are similar to very short straight lines and convert them into line segments in a variety of ways. Motion-based methods estimate line segments from motion vectors in driving scenes using pairs of two consecutive frames. In Step 2, the estimated line segments that tend to converge at the R-VP location are selected by using various constraints. In Step 3, intersection points generated by the lines selected from step 2 are voted to find R-VP. Because the number of intersection points is usually very high, voting algorithms are always used. The most commonly used voting method is the RANSAC-based method.

The accuracy of each method above depends heavily on the type of geometrical features that the method uses. Although the line segment-based methods have achieved high accuracies, they are well-suited only for structured roads. It is known that the edge-based methods, texture-based methods, and motion-based methods can detect R-VPs for both structured roads and unstructured roads. However, texture-based methods cannot be applied to real-time R-VP detection applications because of their high complexity. The edge-based methods are highly sensitive to illuminations due to the character of edge detectors. Eventually, the motion-based methods seem to be the most suitable method to detect R-VPs for both structured roads and unstructured roads because they do not depend on extracting features from the lanes, edges, and texture of the driving scenes. However, the existing motion-based R-VP detection methods for driving scenes still have some limitations as shown in the next sub-section. Therefore, this paper attempts to enhance the performance of the methods.

**Figure 1:**
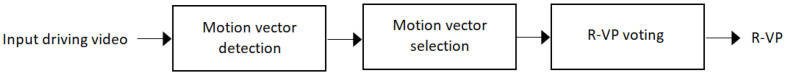
The main processing of existing motion-based road vanishing points (R-VP) detection methods.

In general, various kinds of line segments are estimated from input driving video frames. The line segments that tend to converge at R-VP locations are selected by using various constraints. Because the road surface is not completely flat, the translation of the host vehicles is not completely pure, which causes a small fluctuation of the R-VPs. To compensate for the fluctuation, possible intersection points are firstly found and an intersection point with the highest vote is detected as R-VP by using voting methods such as MLE (maximal likelihood estimator) voting [9], probabilistic voting [10], line-soft-voting [12], cell-based voting [16], and RANSAC-based voting [18,21,22,24]. The next sub-sections summarize the three main processing steps of existing motion-based R-VP detection described in Figure 1.

### 2.2. Motion Vector Detection and Selection

In the motion-based R-VP detection methods [34,35,36,37], motion vectors are used as line segments. Most existing motion-based methods detect a fixed number of corner points in a frame by using corner detectors such as the Shi-Tomasi corner detector [54]. These detected corner points are tracked in the next frame using the Lucas-Kanade method [55]. The corner detection and tracking process are repeated for every pair of two consecutive frames. The corresponding points between two frames form motion vectors. As the sets of detected corner points between frames are often unstable, the estimated motion vectors lead to errors of the R-VP detection. Furthermore, the lengths of the estimated motion vectors are usually short because corner points are tracked only between two consecutive frames. These unstable and short motion vectors make it difficult to detect the FOE of host vehicles with high accuracy.

**Figure 2:**
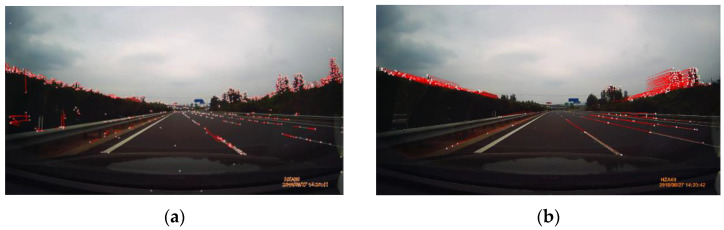
Examples of motion vectors detected from (**a**) two consecutive frames by the existing method and (**b**) several consecutive frames by the proposed stable motion detection method.

Figure 2a shows motion vectors (indicated as red lines) estimated by the existing motion-based R-VP detection methods. The white dots are the heads of the motion vectors. The remaining endpoints are tails. The direction from a tail to a head and the length of a motion vector represent movement information of a corner point. These motion vectors provide a lot of useful information for a wide variety of computer vision applications. However, in the point of view of the R-VP detection problem, these motion vectors still cause some errors for R-VP detection. In detail, most existing motion-based R-VP detection methods assume that longer motion vectors are more convergent to R-VP locations [34,35,36,37]. However, it can be seen in Figure 2a that the short vectors also coincide on the lanes and road boundaries. The short vectors also contribute well to the R-VP detection. These less distinguishable motion vectors do not provide useful information for selecting vectors that are useful for the R-VP detection problem. It is necessary to enhance motion vector detection that facilitates R-VP detection.

The existing motion-based methods attempt to select useful vectors. Motion vectors that are long and not belonging to a certain area where other moving objects exist are selected [34,35,36,37]. In the methods, they eliminate the vectors in the area of on-coming and in-parallel driving vehicles. However, we observe that the motion vector selection also eliminates useful vectors for R-VP detection. It is helpful to keep the motion vectors coming from stationary objects.

### 2.3. Voting Method

In motion-based methods, motion vectors are used as line segments. Because the number of intersections of the line segments is often very high and these lines converge at a small location instead of a single point, a voting process is needed. The RANSAC-based voting has been used commonly for low computational complexity. A minimum number of line segments are randomly selected to generate an estimated R-VP as a hypothesis R-VP. The hypothesis R-VP is voted by the remaining motion vectors. In the existing methods, perpendicular distances between the hypothesis R-VP and every line segment are calculated and used as a metric to vote the hypothesis R-VP. The voting process is applied iteratively for other hypotheses. A hypothesis R-VP that gets the highest vote is detected as R-VP. The distance-based metric does not guarantee to assign the higher scores to the useful vectors (inliers) that tend to highly converge at the R-VP locations. As shown in Section 3.3, the two motion vectors (indicated by the red arrows) have the same perpendicular distance from the hypothesis R-VP (indicated by the blue dot), even though they converge in completely different directions. In this paper, an efficient RANSAC-based voting method is proposed to exclude most of the outliers from the voting process.

## 3. Proposed Method

**Figure 3:**
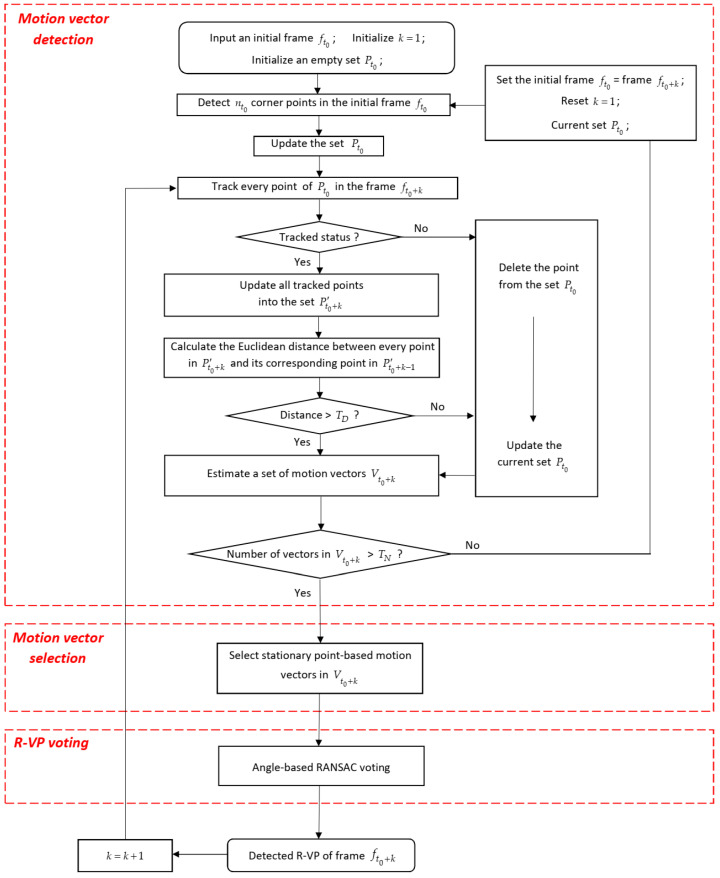
Flowchart of the proposed R-VP detection method.

The proposed method also has the same main processing steps as shown in Figure 1. However, the three processing steps are proposed to improve the robustness and real-time ability of the R-VP detection. The flowchart of the proposed R-VP detection method is shown in Figure 3. The following sub-sections describe three processing steps in detail.

### 3.1. Stable Motion Vector Detection

In contrast to most existing motion-based R-VP detection methods that obtain motion vectors from two consecutive frames, our motion vector detection estimates stable motion vectors over several consecutive frames. From a given first frame $f_{t_{0}}$ at a time $t_{0}$, corner points are detected by using the Shi-Tomasi corner detector [54]. All corner points form an initial set, denoted as $P_{t_{0}}=\left\{p_{t_{0}}\left(1\right),p_{t_{0}}\left(2\right),\ldots ,p_{t_{0}}\left(n_{t_{0}}\right)\right\}$, where $n_{t_{0}}$ is the number of corner points. Every point in the initial set is tracked over the successive frames $f_{t_{0}+k}$ (k=1,2,3,…) by using the Kanade-Lucas optical flow method [55]. Any point in the initial set that is not tracked in the current frame $f_{t_{0}+k}$ is deleted from the set $P_{t_{0}}$. Otherwise, the successive tracked points are updated into the tracked set $P_{t_{0}+k}^{\prime }$. Even though the set is denoted as $P_{t_{0}+k}^{\prime }$, only one set is used and updated for a different time $t_{0}+k$. The initial point $p_{t_{0}}\left(i\right)\in P_{t_{0}}$ and its corresponding tracked point $p_{t_{0}+k}^{\prime }\left(i\right)\in P_{t_{0}+k}^{\prime }$ have the same index i and indicate the pixel-wise coordinates.

Detail procedures to detect stable motion vectors are as follows. If the corner points are not tracked in the current frame $f_{t_{0}+k}$ or have a very short displacement (smaller than a predefined distance $T_{D}$) between the previous frame $f_{t_{0}+k-1}$ and the current frame $f_{t_{0}+k}$, the points in the initial set are deleted. The number of remaining corner points at the frame $f_{t_{0}+k}$ decreases to $n_{t_{0}+k}\left(\leq n_{t_{0}+k-1}\right)$, then the initial set and tracked set are now updated as $P_{t_{0}}=\left\{p_{t_{0}}\left(1\right),p_{t_{0}}\left(2\right),\ldots ,p_{t_{0}}\left(n_{t_{0}+k}\right)\right\}$ and $P_{t_{0}+k}^{\prime }=\left\{p_{t_{0}+k}^{\prime }\left(1\right),\;p_{t_{0}+k}^{\prime }\left(2\right),\ldots ,p_{t_{0}+k}^{\prime }\left(n_{t_{0}+k}\right)\right\}$, respectively. The lifetime of tracked points depends on how fast or slow the movement of the host vehicle is. The corner point tracking process performs iteratively until the number of the remaining points $n_{t_{0}+k}$ is less than a pre-defined threshold number $T_{N}$. Whenever the number of remaining points is less than $T_{N}$, new corner points are detected in the actual frame and appended to the initial set. The new detected corner points and remaining old ones keep a sufficient number of points to estimate stable motion vectors.

The motion vector set $V_{t_{0}+k}=\left\{v_{t_{0}+k}\left(1\right),v_{t_{0}+k}\left(2\right),\ldots ,v_{t_{0}+k}\left(n_{t_{0}+k}\right)\right\}$ is obtained from every remaining point in the tracked set and its corresponding point in the initial set. The length of each motion vector $\left\|v_{t_{0}+k}\left(i\right)\right\|$ is the distance between a point $p_{t_{0}}\left(i\right)$ and its corresponding point $p_{t_{0}+k}^{\prime }\left(i\right)$ at the current frame $f_{t_{0}+k}$. Figure 2b shows that the estimated motion vectors of the proposed method are much longer than the ones taken from two consecutive frames. Compared to Figure 2a, most redundant and short motion vectors are eliminated in Figure 2b. As long motion vectors appear over many successive frames, they are considered to be stable motion vectors. Besides, many long motion vectors coincide with lanes and road boundaries. The stable motion vectors are very helpful to improve the performance of R-VP detection.

### 3.2. Stationary Point-Based Motion Vector Selection

**Figure 4:**
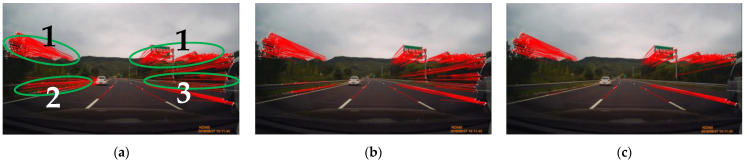
(**a**) Three types of motion vectors in a driving scene, where motion vectors types 1, 2, and 3 come from the stationary background, accelerating, and decelerating vehicles, respectively. The motion vectors (**b**) when applying motion vector type 2 reduction sub-step and (**c**) when applying both motion vector type 2 and 3 reduction sub-steps.

In general, there are three different types of motion vectors in the driving scenes when a camera of a host vehicle moves. The first is motion vectors that are generated from the approaching of stationary objects such as lanes, road boundaries, trees, traffic signs, buildings, etc. Two other motion vectors occur from other moving objects. The second type of vector is generated from other accelerating, overtaking, and land changing vehicles. The third one occurs from on-coming or decelerating vehicles. Figure 4a shows an example of the three types of motion vectors. In this Figure, the numbers 1, 2, and 3 indicate motion vectors from the stationary background, an accelerating vehicle, and a decelerating vehicle, respectively. Type 2 and type 3 motion vectors are numerous and might generate many incorrect R-VPs. Moreover, if the number of moving objects increases, their appearances will affect strongly the accuracy of R-VP detection. To achieve high performance of the R-VP detection, it is important to reduce the motion vectors type 2 and type 3 as much as possible, maintaining type 1.

In the following sub-sections, the proposed stationary point-based motion vector selection method is introduced to reduce two different types of motion vectors respectively, based on the experimental observations.

#### 3.2.1. Reduction of Motion Vector Type 2

It is observed that a current tracked point $p_{t_{0}+k}^{\prime }\left(i\right)$ belonging to motion vector type 2 is closer to the image center when k increases. Then, the motion vector set $V_{t_{0}+k}$ at the time $t_{0}+k$ is updated to maintain motion vectors of which the head points $p_{t_{0}+k}^{\prime }\left(i\right)$ get farther away from the image center $p_{c}$ as follows;

(1) $$V_{t_{0}+k}^{}=\left\{v_{t_{0}+k}^{}(i)\;|\;\left\|p_{t_{0}+k}^{\prime }(i)-p_{c}\right\|>\left\|\left(p_{t_{0}}(i)-p_{c}\right)\right\|,v_{t_{0}+k}^{}(i)\in V_{t_{0}+k}^{}\right\}$$

The above constraint does not guarantee to eliminate all motion vector type 2. It is observed that the motion vector with a completely different direction has equal or very similar $\left\|p_{t_{0}}\left(i\right)-p_{c}\right\|$ and $\left\|p_{t_{0}+k}^{\prime }\left(i\right)-p_{c}\right\|$. For this case, an extended actual head point denoted as $p_{ext\_t_{0}+k}^{\prime }\left(i\right)$ is calculated using Equation (2) and applied to Equation (1) to eliminate efficiently the motion vector type 2.

(2) $$p_{ext\_t_{0}+k}^{\prime }(i)=p_{t_{0}+k}^{\prime }(i)+l\frac{v_{t_{0}+k}(i)}{\left\|v_{t_{0}+k}(i)\right\|}$$

where $p_{t_{0}+k}^{\prime }\left(i\right)$ is extended l pixels along the direction of the corresponding motion vector $v_{t_{0}+k}\left(i\right)$. Figure 4b shows an example of motion vector type 2 reduction where the left side white vehicle-related motion vectors are almost eliminated.

#### 3.2.2. Reduction of Motion Vector Type 3

The motion vector type 3 comes from on-coming or decelerating vehicles. As both motion vector types 1 and 3 have similar directions and lengths, it is difficult to distinguish type 3 from type 1. For such reasons, the region where the type 3 motion occurs frequently is excluded in [34]. The angle between every motion vector and image horizon is used to exclude the region. Motion vectors having an angle in the interval $\left[0^{0},30^{0}\right]$ are excluded in [34]. Our motion vector type 3 reduction uses the angle criterion, but a smaller angle interval is applied to prevent from excluding type 1. Motion vectors with angles in the interval $\left[0^{0},10^{0}\right]$ are excluded from the current vector set. A vector length-based reduction is proposed to reduce the remaining type 3, based on the experimental observation that type 3 tends to be shorter than type 1. A certain percentage of the longest motion vectors are kept in the current vector set $V_{t_{0}+k}$ and others are excluded. In summary, the vectors that have small angles with image horizon or short lengths are eliminated from the current vector set. Figure 4c shows that the right-side truck-related motion vectors are reduced.

### 3.3. Angle-Based RANSAC Voting Method

**Figure 5:**
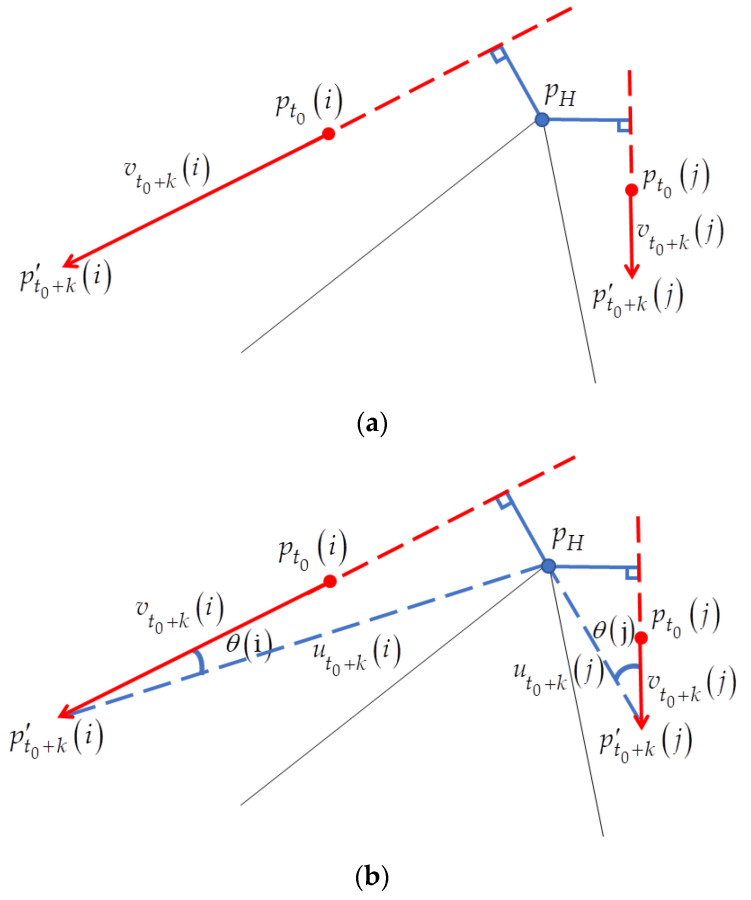
Examples of (**a**) perpendicular distance metric and (**b**) the proposed angle-based metric between vectors and hypothesis R-VP, assuming that hypothesis R-VP $p_{H}$ is obtained from randomly selected two vectors.

In this section, we propose an angle-based RANSAC voting method to overcome the disadvantage of the existing perpendicular distance-based metrics as mentioned in Section 2.3 (Figure 5a). As shown in Figure 5b, the intersection point (indicated by the blue dot) calculated from randomly selected two vectors (indicated by black lines) in the set $V_{t_{0}+k}$ is hypothesis R-VP $p_{H}$. The angle θ(i) between two vectors $v_{t_{0}+k}\left(i\right)$ and $u_{t_{0}+k}\left(i\right)$ is calculated as given in Equation (3) and used for voting. $u_{t_{0}+k}\left(i\right)$ is a vector pointing in the direction from point $p_{t_{0}+k}^{\prime }\left(i\right)$ to the hypothesis $p_{H}$.

(3) $$\theta (i)=\arccos\left(\frac{u_{t_{0}+k}(i)\cdot v_{t_{0}+k}(i)}{\left\|u_{t_{0}+k}(i)\right\|\;\left\|v_{t_{0}+k}(i)\right\|}\right)$$

As the angle θ(i) is smaller when the vector $v_{t_{0}+k}\left(i\right)$ orients closer to the hypothesis, it can be used as a good metric for voting. As shown in Figure 5b, given two vectors $v_{t_{0}+k}\left(i\right)$ and $v_{t_{0}+k}\left(j\right)$ (indicated by the red arrows) have different angles θ(i) and θ(j), unlike the perpendicular distance metric described in Section 2.3 and Figure 5a. Even when two motion vectors move further along their respective directions, the vector $v_{t_{0}+k}\left(i\right)$ orienting closer to the hypothesis has still a smaller angle than another $v_{t_{0}+k}\left(j\right)$. This is the reason why the angle-based metric is helpful to achieve higher performance of the R-VP detection compared to the perpendicular distance-based one. The angle metric is used in the proposed angle-based RANSAC voting method.

A score function is designed by using the exponential function so that a vector with a smaller angle contributes more to the voting. The score S(i) is calculated on every motion vector $v_{t_{0}+k}\left(i\right)\in V_{t_{0}+k}$ for the given hypothesis. A motion vector is regarded as an inlier if it has an angle θ(i) that is smaller than the predefined $T_{\theta }$.

(4) $$S(i)=\{\begin{array}{cc} e^{-\left|\theta (i)\right|} & \theta (i)<T_{\theta }\\ 0 & otherwise \end{array}$$

## 4. Experiments

For the simulations, the proposed R-VP detection is tested on Jiqing Expressway dataset [56] that includes various driving conditions such as straight roads, slightly curved roads, shadows, illuminations, occlusions, moving objects, etc. The dataset is composed of 32 video clips with FHD (1920 × 1080) dimensions. Each video clip has 5393 frames, then the total number of frames is 172,576 frames (=32 clips × 5393 frames). The coordinates of points on the lanes are fully provided in the dataset. Intersection points of the lanes are calculated and used as ground-truth R-VPs.

Two existing R-VP detection methods are also tested for comparison with the proposed method. The motion-based method [34] is implemented as faithfully as possible, which consists mainly of two successive frames-based motion vector detection, region-based motion vector selection, and the perpendicular distance metric-based RANSAC voting. Maximum 500 motion vectors are detected from every two successive frames and used for the voting. The edge-based R-VP method [30] using Canny edge detector and probabilistic Hough Transform is also implemented as faithfully as possible. For the proposed method, 500 corner points are detected in the initial frame $\left(n_{t_{0}}=500\right)$ and tracked. Whenever the number of the tracked points falls under $T_{N}=400$, new 500 corner points will be detected and appended to the initial set. In the stable motion vector detection step, a tracked point is kept when its displacement between two successive frames is bigger than $T_{D}=2$. The parameter $T_{\theta }=45^{0}$ is used to distinguish inliers and outliers.

The performances are evaluated by using the normalized Euclidean distance between the detected R-VP’s coordinate $\left(x_{E},y_{E}\right)$ and the ground truth one $\left(x_{G},y_{G}\right)$. The normalized error dist is given by [34].

(5) $$dist=\frac{\sqrt{{\left(x_{E}-x_{G}\right)}^{2}+{\left(y_{E}-y_{G}\right)}^{2}}}{D}$$

Here D is the length of the diagonal of the image. The error dist ranges over [0,1). When dist is closer to zero, the position of the detected R-VP is closer to that of ground-truth R-VP.

Table 1 shows the accuracy of the proposed method and the other two methods, where the average of the normalized error with its standard deviation is shown. The processing time in milliseconds is also presented for the comparison of computational complexity. To evaluate the validity of the stationary point-based motion vector selection step, the proposed method is tested in four different cases; One is the case of not using motion vector type 2 reduction and type 3 reduction. Two others are the cases of applying either type 2 or type 3 reduction. The last one is applying both reductions.

**Figure 6:**
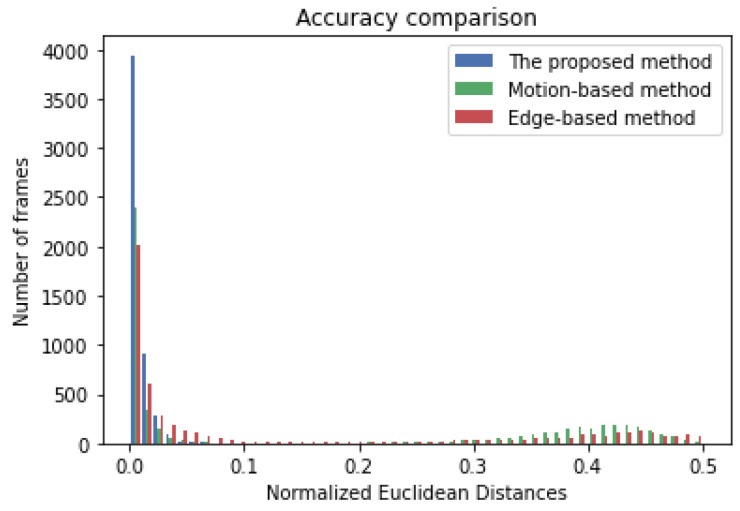
Distribution of normalized error of the proposed (blue), existing motion-based (green), and edge-based (red) R-VP detection methods.

As shown in Table 1, the proposed motion-based R-VP detection, which applies both motion vector type 2 reduction and type 3 reduction, has the highest accuracy (the smallest average error) and the smallest standard deviation. The proposed method has outstanding processing time. The proposed method achieves the average of error 0.0038549 (8.48 pixels in distance) and the standard deviation of 0.0073061 (16.08 pixels in distance). The achieved values are 3.86 and 28.58 times smaller than those of the existing motion-based method, respectively. In particular, the proposed method with a smaller RANSAC iteration number of 45 is the fastest (62.57 ms), maintaining high accuracy similar to the case of iteration number 900. Even in the case of not using the motion vector selection step, the proposed method shows higher accuracy than the two existing methods in terms of both average error and standard deviation. This means that the proposed angle-based RANSAC voting works very well. Simulation results show that the proposed stationary point-based motion vector selection is also efficient to improve the R-VP detection performance. In particular, motion vector type 2 reduction contributes to the improvements slightly more than type 3 reduction. Figure 6 shows the distribution of the normalized errors for the proposed and two existing methods. The normalized errors of the proposed method distribute closer to zero, compared to the others. The performances of the proposed method are also evaluated under three different illumination environments as in Table 2. The video dataset is divided into bright days (17 video clips), slightly dark days (5 video clips), and in-tunnel (10 video clips). The performances are evaluated separately. As shown in the table, the average error and the standard deviation are very similar in three different illuminations. This demonstrates the robustness of the proposed method.

To evaluate the performances according to the feature extractions, the proposed R-VP detections using SIFT (scale-invariant feature transform) and SURF (speeded-up robust features) features instead of the Shi-Tomasi feature are tested and compared. Table 3 shows that both SIFT and SURF have higher accuracies compared to the two existing methods, but the accuracies are lower than those of using the Shi-Tomasi feature. As mentioned in [54], the Shi-Tomasi feature was mainly designed for tracking, whereas SIFT [57] was mainly designed for image matching and SURF [58] is just a speeded-up version of SIFT. Additionally, Table 3 also shows that SIFT and SURF require very high computational costs. Therefore, SIFT and SURF are not efficient features for motion vector estimation in the R-VP detection problem.

We also test the proposed method using the perpendicular distance metric-based RANSAC [59] instead of the proposed angle-based RANSAC. Results in Table 4 show that RANSAC using the angle-based metric is better than that of using the perpendicular distance metric.

**Figure 7:**
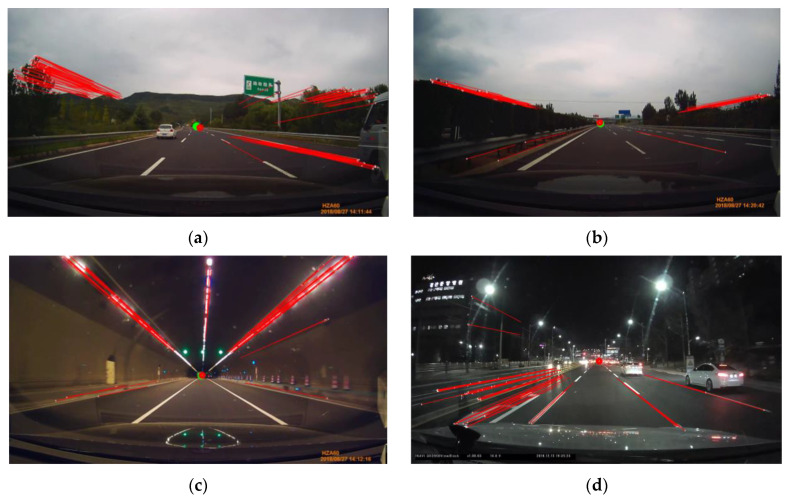
Some examples of the proposed motion-based R-VP detection in various illumination conditions such as (**a**) bright and (**b**) slightly dark daytime, (**c**) tunnel, and (**d**) nighttime. Note that ground-truth R-VP is not indicated in (**d**).

**Figure 8:**
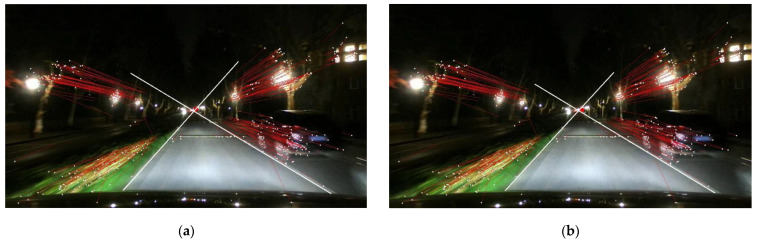
Detected R-VPs (indicated by red circles) and road boundaries drawing by hand (by white lines) under nighttime conditions using the proposed method applying reduction of type 2 and type 3, using (**a**) RANSAC iteration 900 and (**b**) iteration 45.

Some examples of the proposed motion-based R-VP detection are shown in Figure 7, where ground-truth and detected R-VPs are indicated as green and red dots, respectively. The motion vectors are also represented by red lines. Examples show that the proposed method works well in various illumination conditions such as bright and slightly dark daytime, tunnel, and nighttime. An extra test is performed for a nighttime scene as the nighttime video is not included in the ground-truth dataset. Note that ground-truth R-VP is not represented in Figure 7d. We observe that the detected R-VP overlaps visually the intersection point of the road boundaries. The red and green dots in Figure 7a–c almost coincide. Two examples of nighttime R-VP detection are shown in Figure 8, where video frames extracted from “ZJU Day and Night Driving Dataset” [60,61] are used without ground-truth R-VPs. White lines are road boundaries drawing by hand and the red circle is detected R-VP. The red circle and the intersection of road boundaries are also almost coincided. Such coincidences demonstrate the accuracy of the proposed method.

In general, we observed that the proposed method can detect R-VP efficiently when host vehicles move in parallel with road boundaries and road surfaces are almost flat as mentioned in Section 1. The proposed method sometimes fails to detect correctly R-VP in cases of uneven (or bumpy) and curved roads.

## 5. Conclusions

In this paper, we proposed a motion-based R-VP detection method. Three main processing steps often used in the existing R-VP detection methods are carefully examined. Based on the analysis, we propose stable motion detection, stationary point-based motion vector selection, and angle-based RANSAC voting, which are successfully applied to improve the performances of the main three steps.

Through the simulations, we show that the proposed stable motion detection and angle-based RANSAC voting contribute considerably to the improvement of R-VP detection performance. In particular, the proposed angle-based RANSAC voting works much better than conventional perpendicular distance-based voting. The proposed motion-based R-VP detection that combines all three proposed three-step algorithms outperforms the existing motion-based and edge-based R-VP detection methods. The simulation results show that the proposed method is applicable for nighttime, as well as various illumination conditioned driving scenes. It is also shown that the standard deviation of the detected R-VP position is the smallest, compared to others. The performances are very similar in three different illumination conditions. This means the proposed method is robust as well as accurate compared to the existing R-VP detection methods. From the viewpoint of computational complexity, the proposed can be realized in real-time. It is possible to apply selectively the proposed three-step algorithms depending on the system resources. Even though the proposed method is not evaluated on the unstructured road driving video because of the lack of the ground truth datasets, it is expected that it works in a stable on the unstructured road driving.

The proposed R-VP detection method plays an important role to improve the performances of various applications such as online camera calibration, detection of drivable areas, and driving direction of the host vehicle.

## Figures and Tables

**Table 1:** Performance comparisons of the proposed, the motion-based [33] and the edge-based [29] methods in terms of the average distance between the detected and ground truth R-VPs, its standard deviation, and processing time.

Methods			RANSAC Iterations	Average of Error	Standard Deviationof Error	Average of Time Processing (ms)
Edge-based method			900	0.0191347	0.2454778	611.20
Motion-based method			900	0.0148898	0.2088319	147.92
The proposed method	Reduction of type 2	Reduction of type 3				
	✘	✘	900	0.0075473	0.0101119	446.42
	✓	✘	900	0.0049879	0.0080097	410.22
	✘	✓	900	0.0051795	0.0091563	339.21
	✓	✓	900	0.0038549	0.0073061	287.07
	✓	✓	45	0.0042509	0.0073760	62.57

**Table 2:** The performances of the proposed method with reduction of type 2 and type 3 under three different illumination conditions of driving scenes such as bright days, slightly dark days, and in-tunnels.

	Bright Days		Slightly Dark Days		In Tunnels	
	Average of Error	Standard Deviation	Average of Error	Standard Deviation	Average of Error	Standard Deviation
The proposed method with RANSAC iteration 900	0.0041823	0.0072681	0.0041252	0.0073249	0.0032571	0.0073250
The proposed method with RANSAC iteration 45	0.0045382	0.0073146	0.0046241	0.0074313	0.0035902	0.0073825

**Table 3:** The performances of the proposed method with reduction of type 2 and type 3 using scale-invariant feature transform (SIFT) and speeded-up robust features (SURF) features.

Methods	RANSAC Iterations	Average of Error	Standard Deviationof Error	Average of Time Processing (ms)
The proposed method using SIFT	900	0.0091880	0.0100742	1500.37
	45	0.0106574	0.0113037	1335.65
The proposed method using SURF	900	0.0132818	0.0159866	728.19
	45	0.0146137	0.0162612	632.57

**Table 4:** The performances of the proposed method with reduction of type 2 and type 3 using perpendicular distance metric-based RANSAC.

Methods	RANSAC Iterations	Average of Error	Standard Deviationof Error	Average of Time Processing (ms)
The proposed method using distance-based RANSAC	900	0.0093751	0.0136126	283.61
	45	0.0112964	0.0138230	61.82

## Data Availability

Not applicable.
